# Prior balloon valvuloplasty versus DIRECT transcatheter Aortic Valve Implantation (DIRECTAVI): study protocol for a randomized controlled trial

**DOI:** 10.1186/s13063-017-2036-y

**Published:** 2017-07-04

**Authors:** Florence Leclercq, Pierre Robert, Jessica Labour, Benoit Lattuca, Mariama Akodad, Jean-Christophe Macia, Richard Gervasoni, Francois Roubille, Thomas Gandet, Laurent Schmutz, Erika Nogue, Nicolas Nagot, Bernard Albat, Guillaume Cayla

**Affiliations:** 1 Department of Cardiology, University Hospital of Montpellier, Montpellier, France; 2 Department of Cardiovascular Surgery, University Hospital of Montpellier, Montpellier, France; 3 Department of Cardiology, University Hospital of Nimes, Nimes, France; 4 Department of Medical Information, University Hospital of Montpellier, Montpellier, France; 5 Department of Cardiology, Arnaud de Villeneuve Hospital, University of Montpellier, Avenue du doyen Giraud, 34295 Montpellier cedex 5, France

**Keywords:** Transcatheter aortic valve implantation (TAVI), Balloon aortic valvuloplasty, Procedural success, Safety, Randomized clinical trial

## Abstract

**Background:**

Balloon predilatation of the aortic valve has been regarded as an essential step during the transcatheter aortic valve implantation (TAVI) procedure. However, recent evidence has suggested that aortic valvuloplasty may cause complications and that high success rates may be obtained without prior dilatation of the valve. We hypothesize that TAVI performed without predilatation of the aortic valve and using new-generation balloon-expandable transcatheter heart valves is associated with a better net clinical benefit than TAVI performed with predilatation.

**Methods/design:**

The transcatheter aortic valve implantation without prior balloon dilatation (DIRECTAVI) trial is a randomized controlled open label trial that includes 240 patients randomized to TAVI performed with prior balloon valvuloplasty (control arm) or direct implantation of the valve (test arm). All patients with an indication for TAVI will be included excepting those requiring transapical access. The trial tests the hypothesis that the strategy of direct implantation of the new-generation balloon-expandable SAPIEN 3 valve is noninferior to current medical practice using predilatation of the valve. The primary endpoint assessing efficacy and safety of the procedure consists of immediate procedural success and secondary endpoints include complications at 30-day follow-up (VARC-2 criteria). A subgroup analysis evaluates neurological ischemic events with cerebral MRI imaging (25 patients in each strategy group) performed before and between 1 and 3 days after the procedure.

**Discussion:**

This prospective randomized study is designed to assess the efficacy and safety of TAVI performed without prior dilatation of the aortic valve using new-generation balloon-expandable transcatheter heart valves. We aim to provide robust evidence of the advantages of this strategy to allow the interventional cardiologist to use it in everyday practice.

**Trial registration:**

ClinicalTrials.gov identifier: NCT02729519. Registered on 15 July 2016.

**Electronic supplementary material:**

The online version of this article (doi:10.1186/s13063-017-2036-y) contains supplementary material, which is available to authorized users.

## Background

Transcatheter aortic valve implantation (TAVI) is now the standard of care for patients with inoperable severe symptomatic aortic stenosis and an accepted alternative to surgery for high-risk patients [1–6]. The improvement in operator experience and valve technology, combined with better screening and inclusion of lower-risk patients, have resulted in dramatically decreased in-hospital complications after TAVI procedures. However, despite a high procedure success rate (>95%), TAVI has remained associated with complications directly related to the technique (stroke, aortic regurgitation, vascular access bleeding) or to comorbidities frequently associated with aortic valve disease in older and frail patients [3–6]. Reducing periprocedural complications is thereby the key for the future use of TAVI in lower-risk patients. Balloon aortic valvuloplasty (BAV) has been considered as a mandatory step in the TAVI procedure both to facilitate the implantation of the transcatheter heart valve (THV) and to reduce the radial counterforce for optimal device expansion. However, BAV has been shown to carry specific complications and risks [7, 8]. With the development of a new generation of balloon-expandable THVs, associated with low profile and orientable delivery system, the crossing of the valve is facilitated. While both balloon dilatation and the need for post dilatation have been considered to increase the risk of cerebral embolization, avoiding BAV prior to TAVI is attractive and may simplify the procedure [9–12]. Only few nonrandomized studies have shown that direct implantation of the THV is feasible [10, 13, 14], but there are currently no randomized data concerning the safety and efficacy of TAVI performed with new-generation balloon-expandable THVs without prior dilatation of the aortic valve.

DIRECTAVI is the first randomized controlled trial that will evaluate the efficacy and safety of direct implantation of a balloon-expandable new-generation prosthesis, the Edwards SAPIEN 3 THV.

## Methods/design

**Fig. 1:**
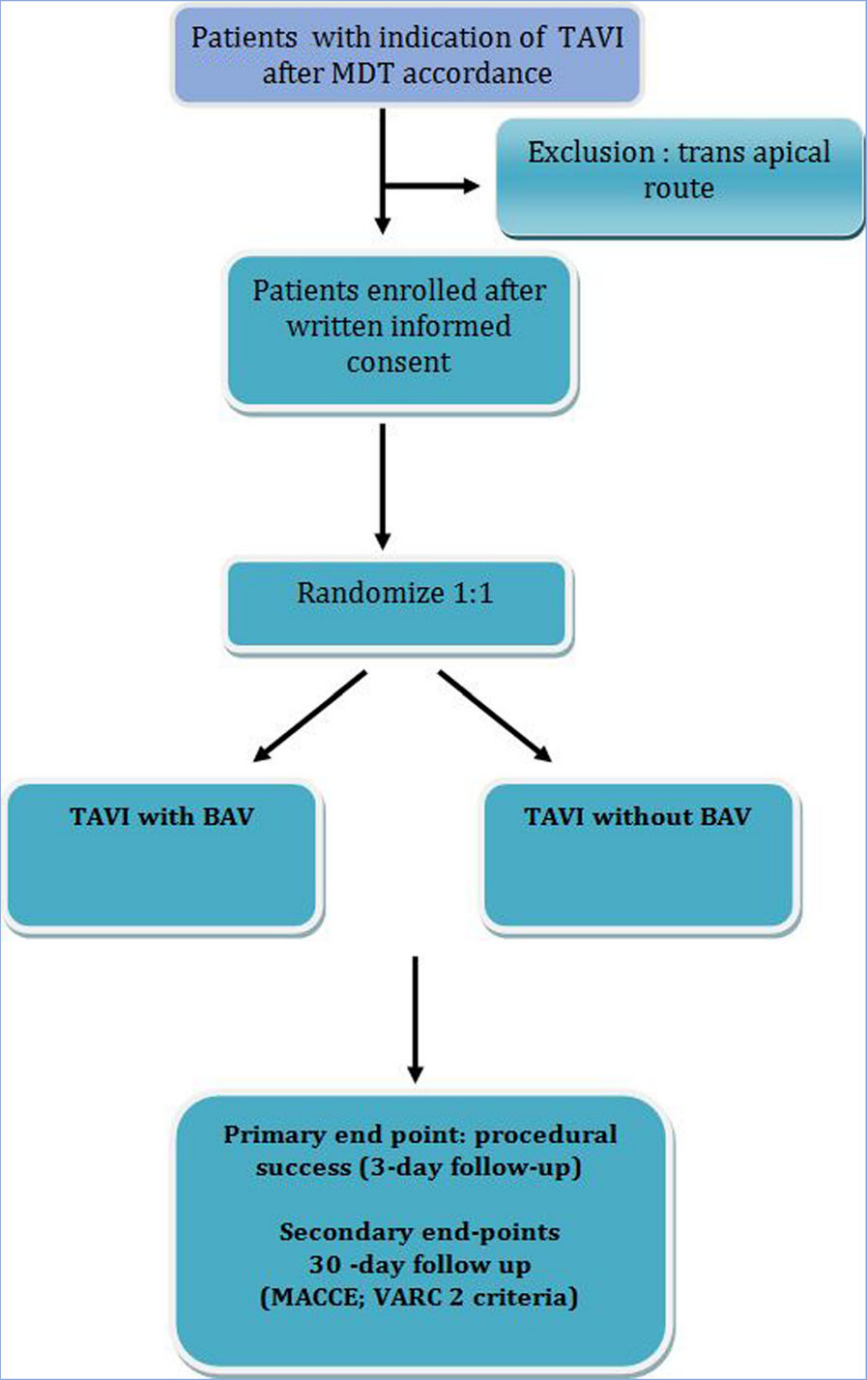
Flow chart of the study. *BAV* balloon aortic valvuloplasty, *MDT* multidisciplinary heart team, *MACCE* major adverse cardiovascular and cerebral events

DIRECTAVI is a prospective, randomized, monocentric, open-label trial that aims to test the hypothesis that TAVI performed without predilatation (test arm) and using the new-generation balloon-expandable Edwards SAPIEN 3 THV is associated with a better net clinical benefit in comparison to procedures performed with predilatation (control arm). The trial has an intentional noninferiority design concerning the primary endpoint. From an ethical standpoint, a sham procedure in the no-dilatation control arm could not be countenanced. The study is conducted in the academic University Hospital of Montpellier, France. The study flow chart is presented in Fig. 1.

### Patient population and procedure

The study population consists of 240 patients who will have TAVI via a transvascular or transthoracic approach. Patients should be deemed eligible for TAVI by a multidisciplinary team including at least an interventional cardiologist, a cardiothoracic surgeon and an anesthetist. All the procedures performed with the third-generation balloon-expandable Edwards SAPIEN 3 THV (Edwards Lifesciences, Irvine, CA, USA) will be considered for inclusion in the study. The SAPIEN 3 THV system is a new generation of balloon-expandable THVs which incorporates various new features to facilitate implantation and minimize vascular injury, stroke, suboptimal positioning, and paravalvular regurgitation [15]. The prosthesis size (23, 26 or 29 mm) and the access route (transfemoral, transcarotid, subclavian or transaortic) are left to the discretion of the operating team. For all patients, both vascular access and aortic valve apparatus are evaluated before the procedure with multislice computerized tomographic angiography (MSCT) of the entire aorta using vascular windows settings. The transfemoral access is the first choice when possible.

Main procedures are done under mild sedation and local anesthesia in our center. Low-profile 14 to 16 French delivery systems are used in all patients with an almost exclusive surgical vascular access. For femoral or carotid access, we use a surgical “preclose technique” in order to avoid arterial cross-clamping and the purse-string effect [16]. For the control group, BAV will be performed with a balloon of 18, 20 or 22 mm diameter according to the THV size. All patients will receive 0.5 mg/kg heparin at the time of introducing the femoral sheath to achieve an activated clotting time of >250 s. All TAVI procedures are performed in the same site by six medical teams comprising at least one interventional cardiologist and a cardiac surgeon, assisted by one nurse for valve-crimping, a nurse who assists the anesthetist and a technician from the catheterization laboratory. A combination of clopidogrel 75 mg and aspirin 75 mg is introduced in all patients after the procedure except in those with an indication for vitamin k antagonists or direct anticoagulant therapy who had only aspirin 75 mg. The vitamin K antagonists or direct orally administered anticoagulants are always stopped at least 2 days before the procedure and usually reintroduced 1 day later.

### Study design

#### Inclusion/exclusion criteria

According to the inclusion and exclusion criteria summarized in Table 1, the study investigators will confirm patient eligibility. All patients who are referred for TAVI in our center and who meet the inclusion criteria will be successively included and randomized. After verification of the eligibility criteria and obtaining informed consent, the patients will be randomized by an investigator to receive either BAV or direct implantation of the valve. For this purpose, the investigator will connect to the secure and dedicated CLINSIGHT website (CS Online) with his username and personal password. The random allocation sequences will be computer-generated by an independent statistician using a 1:1 ratio and permuted blocks of 4 and 6.

**Table 1:** Eligibility criteria for the transcatheter aortic valve implantation without prior balloon dilatation (DIRECTAVI) study

Criteria	Definition
Inclusion criteria	
Patients aged ≥18 years	
Severe aortic valve stenosis	Mean gradient ≥40 mmHg or aortic valve area <1 cm^2^ on TTE^a^
Symptoms suggestive of severe aortic stenosis	Dyspnea, heart failure, angina, syncope
Contraindication for open heart surgery or excessive surgical risk	Decision of the multidisciplinary heart team
TAVI using the balloon-expandable SAPIEN 3 valve	
Vascular access	Transfemoral, transcarotid, transaortic or subclavian access
Exclusion criteria	
Preexisting aortic prosthesis	“Valve in valve” technique
Transapical access	
BAV^b^ performed for less than 1 week	
Recent myocardial infarction	Within the last 3 months
Left ventricular or atrial thrombus	TTE evaluation
Significant mitral or tricuspid regurgitation	>grade II on TTE
Recent cerebrovascular event	Within the last 3 months
Carotid or vertebral arterial narrowing	Stenosis >80% (Doppler or CT scan)
Active internal bleeding	
Thrombocytopenia	Platelet count <50,000/mm^3^
Lack of written informed consent	
Severe mental disorder, drug/alcohol addiction	Specialized evaluation
Life expectancy <1 year	
Participation in another drug or device study	Any study that would jeopardize
	the appropriate analysis of study endpoints
High probability of nonadherence to the follow-up	Social, psychological or medical requirement reason
Pregnancy	

^a^ *TTE* transthoracic echocardiography, ^b^ *BAV* balloon aortic valvuloplasty. *CT* computed tomography

### Data collection and follow-up (see Fig. 2)

**Fig. 2:**
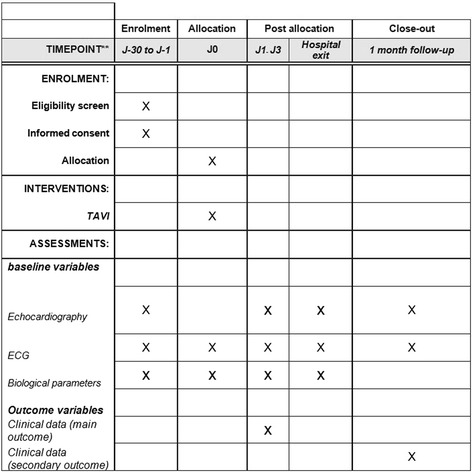
The transcatheter aortic valve implantation without prior balloon dilatation (DIRECTAVI) trial

Baseline data collected from enrolled patients will include demographics, past medical history, previous cardiac investigations and current medication. Patients will undergo transthoracic echocardiography at baseline, post procedure and at 1-month follow-up. Prospective monitoring of adverse and clinical events starts at randomization and continues until 1 month. All major adverse cardiovascular and cerebrovascular events (MACCE) and other serious adverse events will be recorded in the electronic Case Record Form and reported to the coordinating center within 3 days of first identification. On receipt of notification of any adverse or clinical event, the coordinating center will request additional details, specific to the nature of the event. These episodes will be carefully monitored by the trial coordinator and will be part of the information provided at regular intervals to the Clinical Events and Data Monitoring and Ethics Committees. The Clinical Events Committee will be blinded to treatment and will consider each MACCE or adverse event reported and ratify occurrence of an endpoint according to the study definitions.

This 1-month visit is part of the routine practice after TAVI. A consultation appointment will be given to patients when they will be discharged from the hospital, and a dedicated research assistant will call the patients who did not attend on the day after their missed visit. In case of inability for the patient to come, the research assistant and/or investigator will contact the patient GP or cardiologist to obtain some clinical information on the patient. All concomitant care or interventions are permitted during the study follow-up.

### Study endpoints

The primary endpoint, assessing efficacy and safety of the procedure, consists of immediate procedural success defined as the absence of immediate procedural mortality *and* correct positioning of a single prosthetic heart valve into the proper anatomical location *and* intended performance of the prosthetic heart valve (no prosthesis-patient mismatch and mean aortic valve gradient <20 mmHg or peak velocity <3 m/s), *and* no moderate or severe prosthetic valve regurgitation [17].

According to Valve Academic Research Consortium (VARC-2) criteria immediate procedural mortality is defined to capture intraprocedural events that result in immediate or consequent death ≤72 h post procedure [17].

Secondary endpoints include cardiovascular and all-cause mortality, procedure outcomes (length of procedure, incidence of post dilatation, radiation exposure, contrast volume injection), and adverse events (VARC-2 criteria) at 30-day follow-up. Adverse events considered included life-threatening/major/minor bleeding, vascular access complications, acute kidney failure (RANKIN classification stage 2 or 3), pacemaker implantation, neurological events or new hospitalization for cardiac causes. We will also evaluate duration of hospital stay, echocardiography data (transvalvular gradients, aortic regurgitation quantification, left ventricular systolic and diastolic parameters). All the definitions used are in accordance with the VARC-2 guidelines [17].

### Potential biases and prevention

Our study is prone to a number of biases. Since the investigator cannot be blinded during the study, the measurements of the primary outcome components will be made by an independent and blinded cardiologist as follows:

- Absence of immediate procedural mortality: death of the patient during or within 24 h of the procedure
- Correct positioning of a single prosthetic heart valve into the proper anatomical location in the aortic ring
- No prosthesis-patient mismatch (conformity between the size of the prosthesis and the size of the annulus ring) and mean aortic valve gradient <20 mmHg or peak velocity <3 m/s), *and* no moderate or severe prosthetic valve regurgitation. This evaluation using echography and Doppler at 24 and 72 h post procedure will be made by the blinded cardiologist. A potential selection bias will be minimized by the enrollment of all consecutive patients who meet the eligibility criteria

### Ancillary study

An ancillary substudy will enroll 50 patients (25 in each arm) and will be dedicated to evaluate ischemic cerebral events with diffusion-weighted magnetic resonance imaging (DW-MRI) of the brain performed before and after (within 3 days) the TAVI procedure (between days 2 and 5). All consecutive included patients will be proposed to participate in this ancillary study, and all those agreeing to participate will be enrolled until completion of the sample size. MRI will be interpreted by two experienced radiologists blinded to the timing of the imaging and the neurological status of the patient.

### Ethics

The Regional Ethics Committee has approved the trial (Comité de Protection des Personnes Sud Méditerranée, Montpellier, France) and all patients will provide oral and written informed consent. The trial is conducted according to the World Medical Association Declaration of Helsinki and will conform to the ICMJE Recommendations for the Conduct, Reporting, Editing, and Publication of Scholarly Work in Medical Journals. The trial has a Steering Committee, an independent Event Adjudication Committee, and an external Independent Data Monitoring and Safety Committee (DMSC) (registered number: 2015-A01824-45). The DMSC is composed of an interventional cardiologist, a cardiac surgeon, a neurologist, and a statistician. The trial is registered at ClinicalTrials.gov (NCT02729519).

### Statistical consideration

#### Sample size determination

No randomized trials have evaluated the efficiency and safety of direct implantation of THVs without prior BAV. The only randomized study is ongoing and concerns the self-expandable MEDTRONIC CoreValve THV [18]. According to SOURCE, FRANCE 2 AND PARTNER 2 studies [2, 3, 6], we assume a procedural success rate of 95% in the control group. Using a noninferiority threshold of 7%, a power of 80% and 5% significance level, 240 patients must be included to conclude that TAVI without predilatation is noninferior to conventional TAVI.

#### Statistical analysis

A detailed plan of analysis will be elaborated and finalized before the database is frozen (i.e., after completion of the data management). After verification that patients’ characteristics are clinically similar between study arms, the noninferiority of the HFNC device will be assessed by the one-sided Farrington-Manning confidence limit for the risk method using the noninferiority margin of 7%. The noninferiority will be declared if the success rate of the intervention arm (no prior dilatation) will be at most 7% higher than the success rate of the control (prior dilatation) arm. The main analysis will be based on an intention-to-treat analysis whereby all patients randomized in their original arm will be included in the analysis. Given that the primary outcome will be measured during the patient hospitalization, we do not expect any loss to follow-up nor any missing data for the primary outcome. However, any such missing data will be dealt with according to standard approaches, depending on the nature of the missing data [19].

This approach is, therefore, equivalent to the per-protocol analysis recommended for noninferiority trials. Fisher, chi-square and Wilcoxon-Mann-Whitney tests will be used as appropriate to compare secondary outcomes between groups with a superiority approach.

Protocol violations will be reviewed case by case by the investigators to decide whether the patients can remain in the study and in the analyses. For the secondary outcomes, the data of patients who will withdraw or drop out from the study will be retained in the analyses until the time that they leave the study. Statistical significance will be set at 0.05. Statistical analyses will be carried out with SAS (SAS Institute, Cary, NC, USA). The final report will follow the Consolidated Standards of Reporting Trials (CONSORT) 2010 guidelines and the consort extensions for nonpharmaceutical drugs and for equivalence studies.

#### Study management

The DIRECTAVI study is planned to be a randomized open-label trial, conducted and sponsored by the University Hospital of Montpellier (France). Funding has been obtained from Edwards Lifesciences. An Executive Committee composed of experienced clinical investigators will provide trial leadership. A Clinical Events Committee, blinded to the assignment strategy, will adjudicate all clinical events and a DMSC in operation. The committee will comprise physicians who are provided with all the data from medical records necessary to perform optimal adjudication. The study conforms to the Standard Protocol Items: Recommendations for Interventional Trials (SPIRIT) 2013 guidelines (see Additional file 1).

## Discussion

Before deployment of the THV, current medical practice requires right ventricular rapid-burst pacing (rates >180/min) with induction of a functional cardiac arrest for up to 30 s for BAV. This step is thought to be necessary to predilate the native aortic valve and to facilitate an accurate positioning of the valve. BAV is an established palliative procedure for patients with aortic stenosis that has been shown to have numerous detrimental effects [7, 8, 20]: (1) the functional cardiac arrest induced by rapid pacing needed to stabilize the balloon during inflation leads to transient coronary, cerebral, and renal ischemia. In patients with impaired left ventricular ejection fraction, prolonged cardiac depression after rapid pacing may result in hemodynamic failure, (2) causing intraleaflet fractures within calcified nodular deposits of the valve, BAV has been identified as a potential source of embolization of thrombotic and valvular material, (3) due to the displacement of a bulky calcified native valve over a coronary ostium, BAV may increase the risk for coronary obstruction with subsequent myocardial infarction, (4) the local trauma in the left ventricular outflow tract caused by BAV may contribute to conduction disturbances and to permanent pacemaker implantation after TAVI, (5) BAV may induce massive acute aortic regurgitation inducing hemodynamic instability that may require urgent THV placement, (6) with mechanical compression of a “vulnerable area” by calcification, high-pressure BAV may induce annulus rupture, one of the most dramatic and life-threatening complication after TAVI.

A better prosthesis expansion with balloon predilatation, particularly for patients allocated to the self-expandable THV, may in theory reduce the risk of under expansion of the valve and the need for post dilatation. However, the radial force provided by the TAVI device itself is obviously sufficient to guarantee a good expansion in most cases [10, 21]. Lastly, using with contrast injection, BAV may be a help to annular sizing and to evaluate the risk of coronary occlusion in case of lower sinus height. However, with the use MSCT for detailed assessment of the native aortic valve, optimal selection of patients is usually obtained. Conversely, there are potential advantages of avoiding balloon predilatation during TAVI. Firstly, the procedure can be simpler and shorter which can carry advantages particularly in older and frail patients. The radiation dose and the contrast volume are significantly reduced [10, 13, 14]. Reduction in complications associated with BAV (embolization, annulus rupture, conductive disturbances, acute aortic regurgitation) may also be considered [10, 13].

Although recent reports have shown that direct implantation of the valve without balloon predilatation is feasible and yields high success rates, such studies were nonrandomized, registry-type and with relatively small sample sizes [10, 13, 14]. A pilot study was for the first time reported in 2011 by Grübe et al., evaluating the feasibility and safety of TAVI without balloon predilatation in 60 consecutive patients using the self-expanding Medtronic CoreValve prosthesis. The patients were prospectively enrolled in 13 international centers. Technical success rate was 96.7% and post dilatation was required in 16.7% of the patients. In-hospital major events were similar as with the current standard reported approach of TAVI with predilatation. There was no valve embolization. New permanent pacing was needed in 11.7% (7 of 60) of patients [10]. In a monocentric study, Fiorina et al. evaluated 55 consecutive TAVIs performed without predilatation using the self-expandable CoreValve THV. Compared to 45 TAVIs with predilatation performed the previous year, direct TAVI appears feasible and safe regardless of the presence of a bulky calcified aortic valve or the valve size implanted. Device success was higher in direct TAVI, mostly driven by a lower incidence of paravalvular leak [13]. In a retrospective study, Mollmann et al. evaluated 26 consecutive patients undergoing transfemoral TAVI with the Edwards SAPIEN XT prosthesis without predilatation and compared with 30 patients treated previously with predilatation. The procedure was successfully performed in all 26 patients, irrespective of the valve area and the extent of calcification. Post dilatation was required in three patients due to aortic regurgitation >grade 2, and can reduced regurgitation < grade 2 in all cases. Radiation dose and amount of contrast dye were significantly reduced in comparison with the predilatation group. No periprocedural neurological adverse events occurred. Mortality at 30 days was 0% [14]. The procedure appears safe and feasible even with self-expanding THVs which are able to “dilate” the stenotic aortic valve through the radial forces of the self-expanding nitinol frame. Post dilatation is, however, frequently required with this device [13, 21]. Chan et al. reported two cases in which balloon predilatation was not performed initially during TAVI but eventually required to facilitate device crossing and implantation. They illustrated the importance of case selection and drew attention to the potential limitation in performing TAVI without balloon predilatation which is not always feasible [22]. More recently, a meta-analysis of 18 studies incorporating 2443 patients showed that no balloon predilatation prior to TAVI was safe and feasible and associated with fewer complications and short-term mortality in selected patients, especially using the self-expandable valve [23].

Direct comparative studies of patients receiving TAVI, with or without prior BAV, with new-generation devices are lacking. In a recent nonrandomized study, Bijuklic et al. reported a significantly higher volume of cerebral ischemic lesions on cerebral magnetic resonance imaging (MRI) after implantation of a balloon-expandable aortic valve without prior BAV. The authors speculated that predilatation leads to plaque fragmentation which can reduce the risk of embolization and the size of pieces that embolize during stent implantation. In that study, however, most patients undergoing TAVI with the Edwards SAPIEN 3 THV had no BAV and they were compared with a historical control group of patients who received either an Edwards SAPIEN XT or an Edwards SAPIEN 3 [18]. No difference in MACCE was also reported by Pagnesi et al. in a cohort of 517 patients undergoing transfemoral TAVI with various generation devices with or without pre-BAV but the rate of post dilatation was increased in the group without prior valvuloplasty [24]. We recently presented in a pilot study the rate of embolic stroke evaluated with MRI in 46 consecutive patients undergoing TAVI with the balloon-expandable EDWARDS SAPIEN 3 THV with or without balloon predilatation. Our results did not show significant differences in cerebral ischemic lesions between the two groups and the new lesions were mainly lacunar [25].

The ongoing SIMPLIFy study randomizes 110 patients with LVEF ≤35% to TAVI without BAV (experimental group) or TAVI with BAV (control group) with the safe expandable Medtronic Corevalve THV [26]. The primary composite efficacy endpoint will include all-cause mortality, stroke, nonfatal myocardial infarction, acute kidney injury, or pacemaker implantation at 30-day follow-up.

Although this study will be implemented in a single hospital, which may limit the generalization of the findings, the design of the DIRECTAVI trial is unique in that no randomized comparisons have been made between TAVI performed with or without predilatation of the aortic valve using the third-generation SAPIEN 3 THV. While direct implantation of the THV is probably associated with reduction of procedure duration and radiation exposure, we do not know if this technique will be associated with net clinical benefits. Of particular concern is the possible increase of risk of stroke associated with direct implantation of the THV [18, 24]. Feasibility of the technique (noninferiority study) has also to be demonstrated. Simplifying the procedure and reducing complications of TAVI is challenging considering the future extension of the procedure to intermediate and low-risk patients [27]. Finally, the findings of DIRECTAVI will help to define the optimum strategy of the TAVI procedure and will facilitate evidence-based guidelines on the controversial issue of whether to predilate or not predilate the valve before implanting the THV.

## Trial status

This study has been recruiting patients since May 2016.

## Additional file

Additional file 1: SPIRIT 2013 Check list. (DOCX 22 kb)

## Abbreviations

BAV: Balloon aortic valvuloplasty
DIRECTAVI: Direct transcatheter aortic valve implantation
DMSC: Data Monitoring and Safety Committee
DW-MRI: Diffusion-weighted magnetic nuclear imaging
MACCE: Major adverse cardiaovascular and cerebral events
MSCT: Multislice computed tomography
TAVI: Transcatheter aortic valve implantation
THV: Transcatheter heart valve
VARC: Valve Academic Research Consortium
